# Potential association of prolonged patient interval and advanced anatomic stage in breast cancer patients in the area affected by the 2011 triple disaster in Fukushima, Japan: Retrospective observational study

**DOI:** 10.1097/MD.0000000000026830

**Published:** 2021-08-13

**Authors:** Akihiko Ozaki, Sawano Toyoaki, Manabu Tsukada, Yuki Shimada, Ayumu Kawamoto, Ji-Wei Wang, Divya Bhandari, Masaharu Tsubokura, Hiromichi Ohira

**Affiliations:** a Research Center for Community Health, Minamisoma Municipal General Hospital, Minamisoma, Fukushima, Japan; b Department of Breast Surgery, Jyoban Hospital of Tokiwa Foundation, Iwaki, Fukushima, Japan; c Department of Surgery, Jyoban Hospital of Tokiwa Foundation, Iwaki, Fukushima, Japan; d Department of Radiation Health Management, Fukushima Medical University School of Medicine, Fukushima, Japan; e Department of Surgery, Minamisoma Municipal General Hospital, Minamisoma, Fukushima, Japan; f Department of Neurosurgery, Minamisoma Municipal General Hospital, Minamisoma, Fukushima, Japan; g Faculty of Medicine, University of Szeged, Szeged, Hungary; h Key Lab of Health Technology Assessment of Ministry of Health, School of Public Health, Fudan University, Shanghai, China; i Medical Governance Research Institute, Minato-City, Tokyo, Japan.

**Keywords:** breast neoplasms, Fukushima nuclear accident, help-seeking behavior, neoplasm staging

## Abstract

For five years after the 2011 triple disaster (earthquake, tsunami, and nuclear disaster) in Japan, the proportion of patients with undiagnosed symptomatic breast cancer remained elevated in the coastal area of Fukushima. These individuals experienced a prolonged interval from first symptom recognition to initial medical consultation (hereafter referred to as the *patient interval*). We aimed to investigate how this prolonged patient interval affected disease staging.

Using patient records, we retrospectively extracted females with newly and pathologically diagnosed breast cancer who initially presented to Minamisoma Municipal General Hospital from March 2011 to March 2016. We estimated the proportion with advanced-stage disease (III, IV) according to the patient interval duration (<3 months, 3–12 months, and 12 months plus). A cut-off patient interval value was determined based on the previous evidence with regards to impacts on survival prospects. Logistic regression approaches were used to fulfill the study outcome.

The proportion of patients with advanced-stage disease was 10.3% for < 3 months (7/68), 18.2% for 3–12 months (2/11), and 66.7% for more than 12 months (12/18). We found a similar trend using the multivariate logistic regression analyses.

Prolongation of the patient interval was associated with advanced-stage disease among female patients with breast cancer.

## 1 Introduction

Early diagnosis and treatment are the cornerstones of breast cancer management. Although awareness programs have led to a high uptake of breast cancer screening in high-income countries such as the US^[1]^ and a majority of European countries,^[2]^ a large proportion of breast cancer patients are diagnosed only after they recognize relevant symptoms (e.g., breast lumps and/or nipple discharge). In Japan, 51.7% of the patients newly diagnosed in 2017 presented with symptoms suggestive of breast cancer.^[3]^ Further, some of these patients put off their first medical consultation even when they recognized their symptoms.^[4,5]^ While reasons for these delayed presentations should be explored individually, various patient-level and external factors are reportedly associated with this phenomenon.^[4,6,7]^

Among these external factors, disasters and crises have become much more prominent as the recent coronavirus (COVID-19) pandemic could potentially shift priorities onto individuals with more urgent symptoms than those suggestive of breast cancer.^[8]^ Indeed, a similar phenomenon occurred during the 2011 triple disaster in Japan (earthquake, tsunami, and the Fukushima Daiichi Nuclear Power Plant Disaster); in the So-so District, the northen coatral area of Fukushima, Japan, which houses the power plant, the proportion of undiagnosed symptomatic breast cancer patients who experienced prolonged patient intervals (the period from first symptom recognition to initial medical consultation) remained elevated for five years after the disaster when compared to the pre-disaster baseline.^[9]^ One major plausible reason for this phenomenon is fluctuating social and family support due to mass evacuations following the disaster.^[9–12]^ However, little is known about how this prolonged patient interval has influenced survival prospects. In breast cancer patients, anatomic staging has been an important surrogate marker of survival prospects despite the recent emergence of prognostic staging,^[13]^ and this is also the case with recent Japanese breast cancer patients.^[14]^ Therefore, we evaluated how a prolonged patient interval affected anatomic staging among post-disaster undiagnosed symptomatic breast cancer patients in the area affected by the 2011 Fukushima triple disaster.

## 2 Methods

### 2.1 Study setting and participants

This is a secondary analysis of our previously published work that retrospectively compared the patient interval between pre- and post-disaster undiagnosed symptomatic breast cancer patients in the So-so District, Fukushima.^[9]^ We considered all 97 post-disaster breast cancer patients who were included in our previous study. In short, all patients were women with breast cancer diagnoses based on pathological findings, and all of them had continuously lived in the So-so District before and after the disaster. These patients initially presented to Minamisoma Municipal General Hospital (MMHG), located in Minamisoma City, the largest municipality in the So-so District, Fukushima, from March 11, 2011 to March 10, 2016.^[9]^ In our previous work, we considered Watanabe Hospital, which is also in Minamisoma City, as the other study setting, but we excluded it in the present analysis due to its discontinuation of inpatient functions after the disaster.^[9]^

### 2.2 Analytical data

We retrospectively collected the sociodemographic and clinical characteristics of the patients from medical records stored in the MMGH, which were summarized in our previous study.^[9]^ Following our previous study^[9]^ and other evidence on associations between patient interval and survival prospects of breast cancer,^[4,15–17]^ we categorized patient intervals into three groups: less than three months, three to twelve months, and twelve months or more. In general, patient intervals of three months or longer are believed to impair survival prospects of breast cancer patients.^[15,16]^ Further, a patient interval of twelve months or longer can be observed in resource-limited settings such as in disaster aftermaths^[9,11]^ and low- and middle-income countries, with extensive impacts on survival prospects of breast cancer patients.^[4,15,17]^

We diagnosed the patients according to anatomic staging as defined by the American Joint Committee on Cancer Eight Edition^[18]^ and categorized them into two groups: advanced stage (Stage III, IV) and otherwise (Stage 0, I, or II). Categorification of other variables mostly followed our previous study.^[9]^

### 2.3 Data analysis

For this study, we performed two analyses. First, we estimated the proportion of patients diagnosed with advanced cancer in the three groups according to length of patient interval and evaluated whether there was a trend of increased proportion of advanced cancer in these three groups with the Stata command “*nptrend* ”. Second, we constructed a logistic regression model with length of patient interval as the primary independent variable and advanced stage of cancer as the dependent variable. We also considered other sociodemographic and clinical characteristics as adjusting variables, using the backward stepwise variable selection method (inclusion criteria, *P* < .1). We used Stata IC15.0 to conduct these two analyses. We obtained ethical clearance from the Ethics Committee at MMGH.

## 3 Results

A flow of patient selection and key patient characteristics are described elsewhere.^[9]^ In summary, of 131 patients who visited the study site during the study period, 97 were symptomatic patients (50 or younger: 16 (16.5%), 51–64: 33 (34.0%), older than 65: 48 (49.5%)), and all of them were considered in the following analysis. Of the 97 patients, the proportion of patients with a patient interval of less than three months, three to twelve months, and twelve months or above was 70.1% (68), 11.3% (11), and 18.6% (18), respectively. Similarly, the proportion of anatomic stages 0, I, II, III, and IV was 5.2% (5), 29.9% (29), 43.3% (42), 14.4% (14), and 7.2% (7), respectively.

**Figure 1:**
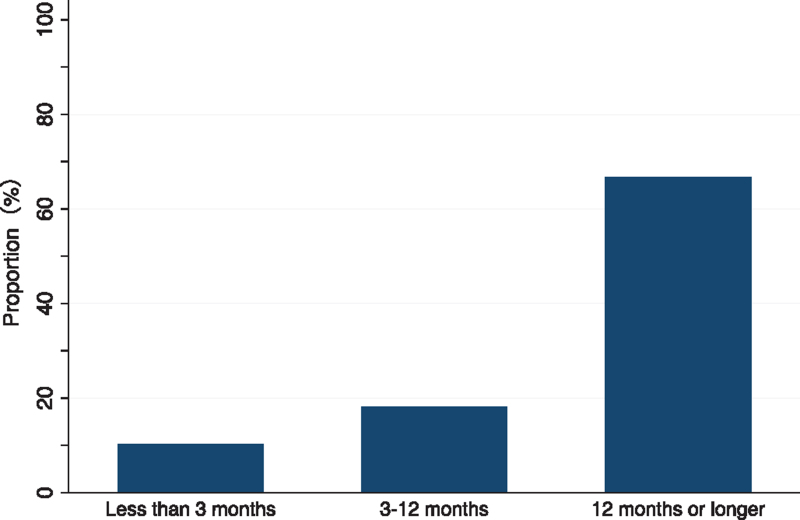
A proportion of advanced cancer according to the length of patient interval among the post-disaster undiagnosed symptomatic breast cancer patients.

Figure 1 shows the association between patient interval and advanced stage diagnosis. The proportion of breast cancer patients diagnosed with advanced stage disease was 10.3% (7/68), 18.2% (2/11), and 66.7% (12/18) in patients with a patient interval of less than three months, three to twelve months, and twelve months or longer (*P* for trend < .001), respectively.

Table 1 shows the findings of univariate and multivariate logistic regression analyses for advanced cancer. Adjusting for other covariates, the patients with a patient interval of twelve months or longer had higher odds of being diagnosed with advanced cancer when compared to those with a patient interval of less than three months (odds ratio: 22.77; 95% confidence interval: 5.19–99.82; *P* < .001).

**Table 1:** Univariate and multivariate logistic regression analyses for diagnosis of advanced cancer.

	Univariate analysis		Multivariate analysis	
Variable	Odds Ratio (95% CI)	*P*-value	Odds ratio (95% CI)	*P*-value
Length of patient interval				
Less than three months	1.00			
Three to twelve months	1.94 (0.35–10.82)	.45	1.06 (0.18–6.19)	.95
Twelve months or longer	17.43 (4.97–61.07)	<.001	22.77 (5.19–99.82)	<.001
Distance from cancer center	1.01 (0.93–1.09)	.90		
Referral from other medical providers				
No	1.00			
Yes	0.73 (0.25–2.09)	.55		
Days from initial medical consultation to first examination	1.00 (1.00–1.00)	.22		
Residential address at the timing of the 2011 triple disaster				
Non--evacuation zone of So-so District	1.00			
Voluntary evacuation zone	0.75 (0.21–2.69)	.66		
Mandatory evacuation zone	0.55 (0.08–3.68)	.54		
Age				
–50]	1.00			
(50–65]	1.85 (0.42–8.12)	.42		
(65–	2.11 (0.52–8.53)	.29		
Engaged in full-time job				
No	1.00			
Yes	0.76 (0.23–2.54)	.65		
Number of family members				
0–1	1.00			
2–3	0.48 (0.15–1.50)	.20		
More than 4	0.76 (0.18–3.14)	.70		
Living with a partner				
No	1.00			
Yes	1.10 (0.40–3.06)	.85		
Living with children				
No	1.00			
Yes	0.36 (0.13–1.03)	.06		
Symptom realization of lump				
No	1.00			
Yes	1.41 (0.16–12.76)	.76		
Hormone receptor				
Negative	1.00			
Positive	0.42 (0.09–1.94)	.27		
ASA physical classification system				
Normal healthy patient	1.00			
Patient with mild systemic disease	1.08 (0.38–3.07)	.88		
Patient with severe systemic disease	1.71 (0.36–8.15)	.50		
Body mass index (kg/m^2^)				
–25]	1.00		1.00	
(25–30]	0.19 (0.05–0.76)	.02	0.17 (0.03–0.96)	.045
(30–	0.81 (0.25–2.63)	.72	1.40 (0.31–6.24)	.66
History of benign breast disease				
No	1.00			
Yes	1.89 (0.32–11.14)	.48		
Mammography screening within two years				
No	1.00			
Yes	0.69 (0.14–3.45)	.66		
Family history of any cancer				
No	1.00			
Yes	0.47 (0.17–1.31)	.15		

## 4 Discussion

In this study, we found that prolonged patient intervals was associated with advanced stage diagnosis among symptomatic but undiagnosed breast cancer patients in the area struck by the 2011 Fukushima triple disaster. In particular, in patients with a delay of 12 months or longer, two-thirds of the patients were diagnosed with advanced cancer. This result is consistent with previous studies,^[16]^ demonstrating the importance of timely medical consultation even in the aftermath of disasters and/or other crises.

This observation has significant clinical implications, given the findings of our previous study, which analyzed associations between prolonged patient interval and advanced-stage diagnosis among breast cancer patients in the same setting following the triple disaster.^[19]^ Surprisingly, the proportion of patients with advanced cancer decreased as the interval from initial medical consultation to treatment inception was prolonged, which is totally opposite from the present finding. A plausible explanation for this difference is the involvement of healthcare professionals in the process following the initial medical consultation. Namely, healthcare professionals can triage new breast cancer patients and start treatment, particularly for those with urgent medical conditions.^[19]^

In this regard, it is imperative to further enhance awareness of breast cancer symptoms among undiagnosed symptomatic breast cancer patients and the general public so that patients can accurately realize the severity of their symptoms and so that family members and friends can urge them to schedule their initial consultation. To this end, we believe it is necessary to combine both a population approach and a high-risk approach. For the population approach, it is important to motivate media outlets and local municipalities to prepare articles and programs to raise awareness about the importance of timely medical consultation in breast cancer. We lobbied various media outlets when our previous paper^[9]^ showed an increased delay in medical consultation among breast cancer patients after the disaster, and it was featured in numerous newspaper articles in Japan. For the high-risk approach, it is important for community health workers to individually approach women with reduced family and social support.

There are several limitations in this study, including small sample size, single disaster and institutional assessment, and use of surrogate outcomes of survival prospects. However, it is often difficult to conduct a large-scale study with robust outcome measures in disaster aftermaths. Further, every disaster is unique, and generalizability is a concept that is difficult to achieve in disaster studies. In this regard, it is important to accumulate small-sized evidence on various health risks after disasters.

In conclusion, a delayed initial medical consultation was associated with advanced stage diagnosis among patients with undiagnosed symptomatic breast cancer in the area affected by the 2011 triple disaster in Fukushima, Japan. To mitigate health issues, both population and high-risk approaches should be combined, and awareness of timely medical consultation should be further enhanced, in and beyond the area affected by the 2011 Fukushima triple disaster. The health implications of interrupted cancer care have become apparent during the current COVID-19 pandemic.^[20]^ The cancer lessons of the 2011 Fukushima triple disaster should be widely shared in the global medical and public health communities.

## Author contributions

**Conceptualization:** Akihiko Ozaki, Sawano Toyoaki, Manabu Tsukada, Yuki Shimada, Ayumu Kawamoto, Jiwei Wang, Divya Bhandari, Masaharu Tsubokura, Hiromichi Ohira.

**Writing – review & editing:** Akihiko Ozaki, Sawano Toyoaki, Manabu Tsukada, Yuki Shimada, Ayumu Kawamoto, Jiwei Wang, Divya Bhandari, Masaharu Tsubokura, Hiromichi Ohira.
